# A rather wild imagination: who is and who is not a migrant in the Czech media and society?

**DOI:** 10.1057/s41599-022-01240-2

**Published:** 2022-07-06

**Authors:** Monika Gabriela Bartoszewicz, Otto Eibl

**Affiliations:** 1 Department of Technology and Safety, UiT The Arctic University of Norway, Tromsø, Norway; 2 Department of Political Science, Masaryk University, Brno, Czech Republic

**Keywords:** Politics and international relations, Cultural and media studies

## Abstract

This paper focuses on migrants and migration in the context of the Czech Republic, an ethnically and nationally homogeneous country without significant migration experience. Despite this fact, the issue of migration became very prominent in 2015 and has been an integral part of Czech political and public discourse since then. Although the topic has attracted scholarly interest, but the reflection on migrant images held by citizens has been omitted. To fill this gap, first, we conducted a quantitative computer-assisted content analysis of the main Czech media (2015–2018) to investigate how important the issue of migration was and in what context migrants and migration were discussed in the media. We then conducted a series of focus groups with Czech citizens to answer not only how they perceived migrants and migration in general, but also how they perceived the (quality of) media coverage of this issue. The findings offer insight into patterns of media consumption: Our respondents were well aware that media representation of the topic is exaggerated and does not include all possible points of view. The prevailing perception was that the mostly negative media representation was fixated on the image of a migrant coming from the Middle East, most likely to be a terrorist who is not going to adapt to a “normal” life in the Czech Republic. Indeed, the very term migrant is mainly associated with someone who, according to the mental projections of the respondents, is “different” at first sight, fails to fit in and integrate into the majority society, does not look for work thus becoming dependent on the social system of the host country. In other words, for Czechs, people who come to settle and work are excluded from the socially constructed category of migrants.

## Introduction

Although migration is a natural process and a permanent feature of the human condition, it very often turns into an emotionally charged and sometimes very controversial political issue that can divide entire societies. This was demonstrated after 2015, when there was a rather intense migration to Europe—whether it was from the Middle East or from North Africa. Either way, the issue received a huge amount of media interest and attention from political elites. The progress of the migrants was also followed with great interest (and concern) by the citizens of individual EU countries.

This was no different in the Czech Republic. Despite the fact that the issue of migration became omnipresent and in the political sphere (or perhaps because of it), the issue is often used as a powerful emotional trigger (Boccagni and Baldassar, 2015; Albrecht, 2016) and effective mobilization tool (Jeandesboz and Pallister-Wilkins, 2016; Majtényi et al., 2019). Indeed, it was used if not in all then in most of the subsequent political campaigns after 2015. Thus, migration is still a salient issue in the public debate in the Czech Republic.

In our paper, we are interested in the relationship between Czech media representation of migration and migrants from 2015 to 2018 and how Czechs perceive them. Our research builds on a relatively large body of work on a similar topic. Amid the scholarship on workers of immigrant origin (Drbohlav, 2002, 2003; Drbohlav and Janská, 2009), there is a group of authors who, via case studies, have taken a closer look at trends connected with selected foreign national and ethnic groups and traced diverse variables such as their motives, occupations, status, or living conditions (Horáková et al., 2001; Hulíková and Kocourek, 2005; Černík et al., 2005; Černík, 2006). Their work exposed the role of various intermediary actors such as recruitment agencies and visa traders, often operating within legal loopholes or complex and illegal networks (Kroupa et al., 1997; Trávníčková et al., 2004; Nekorjak, 2006). Recent work has focused on second-generation migrants; notions about belonging and the uncertain future have been detected in this group (Souralová, 2014; Cheng and Hu, 2015; Janská and Bernard, 2018). At times, the Czech studies have gone beyond the host country’s borders by conducting research in countries of origin, where the role of remittance inflow is emphasized (Hamar and Szaló, 2007). In this context, the most recently emerging group of authors has been evaluating national-level policies and their impacts on the issue of immigration (Uherek, 2004; Jelínková and Szczepaniková, 2008; Klásková and Císař, 2020).

While we engage with existing research that focus on Central and Eastern Europe (e.g., Kluknavská et al., 2021; Navrátil and Kluknavská, 2022; Kovář, 2022), we consider our decision to combine data from the media with data from focus groups, in which we reflected not only respondents’ relationships to migration (and ideas about it), but also how they perceived media reality and media content in general, to be significantly innovative.

Subsequently, to understand the dynamics between migration discourse conceptualized as communicative acts linked with the exercise of power (Holzscheiter, 2005) and society’s attitudes and feelings about migration, we carried out a series of focus group interviews (FGIs). We have followed the path suggested by Scheufele’s analytical model: investigating how frame building shapes the audience frames as the dependent variable, to find the explicit and direct, individual-level consequences of framing (2000, pp. 306–308). A Dutch study has shown that the more news media reported about immigration-related topics, the higher the aggregate share of vote intention for anti-immigrant parties, even when controlling for real-world developments (Boomgaarden and Vliegenthart, 2007). This shows that frames guide the processing of information and related forming of attitudes and judgments, cognitive responses, as well as attitudes and beliefs in individuals (Igartua and Cheng, 2009). Cultivation analysis looks at how frame-valence influences attitudes towards immigration and the influence of media framing from a long-term perspective with a repeated and sustained exposition over time (Potter, 1993, 2014; Romer et al., 2014). This is important in the context of our study, as we aim to unravel the media determinants of people’s preferences regarding migration policy, their expectations of immigrants, and economic concerns with a wider time margin, going beyond the immediate “Kodak moment” of 2015.

In what follows, we present the results of a content and framing analysis of the main Czech media between 2015 and 2018 and the main outcomes of a series of focus group interviews conducted in 2020. In both cases, the focal point is migration and the “image” of migrating individuals. Whereas the first part of this paper gives an overview of the main issues or frames related to this issue and the salience of the topic, the latter part gives insight into the minds of “average” Czech citizens. Accordingly, in our research we were driven by the following questions: How intense was the coverage of the migration issue in the Czech media? Specifically, in what contexts were migrants and migration discussed in the media? What were the prevailing frames of the media coverage? And subsequently, what does the Czech public think about migration and how do citizens perceive and understand the issue of migration? The first part of our research is based on computer-assisted quantitative content analysis and provides a more descriptive view of the issue coupled with the analytical outcome of media analysis. The second part of the research works with data from focus groups and offers a detailed view of how migration is perceived.

The quantitative analysis of media images does not bring any significant surprises: The attention of the media culminated with the migration wave in 2015. In the following years, the issue was still salient and had some frame-dependent attention peaks. Reporting became slightly less intensive (in terms of the gross number of published articles), while simultaneously the focus of journalists also changed slightly over the course of time. At first they provided the big picture, but in the end they paid attention to more thematically narrow stories.

However, the qualitative take on socially constructed images brings a colourful view of the migration phenomenon. We are able to contextualize how exposure to media messages augmented by the dynamic of the social identity theory (Tajfel and Turner, 2004) allow to understand how people distinguish between migrants. Furthermore, we offer novel insights into the currently valid image of the migrant, which de facto includes only people who are threatening the host society by their inability and unwillingness to integrate. In contrast, individuals who come in order to work and who our respondents indicated as culturally and ethnically close to Czech people, are not considered migrants.

### The 2015 Migration Crisis: a short introduction to the Czech case

After the democratic transition in the late 1980s and early 1990s, the countries of Central and Eastern Europe (CEE) started to gradually shift from transit to target destinations (Bartoňová, 2002). The most recent data from the Ministry of the Interior (2020) indicate that the number of foreigners in the Czech Republic continues to rise despite the pandemic. As of 31 December 2021, a total of 634,000 foreign nationals, i.e. roughly 6% all Czech residents were registered in the Czech Republic. Twenty-six per cent of all foreigners in the Czech Republic were Ukrainians. Other significant nationalities were Slovaks, Vietnamese, Russians, Germans, and Poles. When it comes to illegal migration, although its share in the total number of migrants is not significant (and the Czech Republic is only a transit country for many migrants), detected cases of illegal migration are very often reported in the media, and the average citizen may have the impression that this is serious problem. A total of 7000 irregular migrants were detained in Czech Republic in 2021 including 2690 in the last quarter alone (most often they are Afghan, Syrian, Moroccan, or Turkish) (Ministry of Interior, 2021).

The Czech Republic’s limited experience with immigration can be divided into three historical periods of migration policy (Baršová and Barša, 2005). The first was between 1900 and 1996, when migration was not restricted. Subsequently, in the period 1996–1999, the conditions for entry of migrants were tightened, primarily due to preparations for European Union accession, increased unemployment, and the impact of illegal migration. The third period, from 1999 to the present, has seen efforts of the Czech state and its leaders to approach migration more comprehensively. The Czech Migration Policy Strategy (*Strategie migrační politiky České republiky*), approved in 2015, is an essential document shaping Czech migration policy that set the legal stage at the onset of the migration crisis. Its key message is that the state seeks to promote legal migration that can benefit Czech citizens, e.g., by filling job vacancies.

At the level of the European Union, we can see great efforts by the Czechs to preserve the autonomy of the nation state in deciding about forms of legal migration. Unsurprisingly, the main legislative regulation of migration policy in the Czech Republic is the Aliens Act, which determines the “conditions of entry to Czech Republic, stay and departure from the territory of the foreigner” (Law Number 326/1999 Coll.).

The 2015 migration crisis was not characterized in the Czech Republic by a huge number of incoming refugees crossing the border and applying for protection. Rather, the crisis was largely felt in the meta sphere of public debates and media coverage. In each country in Central Europe the primary response focused heavily on securitization of the issue with considerable criticism directed against the proposals put forward by the EU and Germany and calls to reject them (Bartoszewicz, 2016, 2019). In the Czech Republic, anti-establishment and populist parties (e.g., the Freedom and Direct Democracy Party) gained a new foothold, anti-immigration rhetoric became normalized in political debates and even mainstream politicians adopted very strong anti-migration positions. As a result, the public debates centred on controlling migration and greater selectivity of immigrants. There has been an emphasis on resisting compulsory quotas for relocation and resettlement of refugees, supporting more robust border protection, and enhancing humanitarian aid directly in conflict zones.

Simultaneously, social attitudes toward migrants and refugees have trended in an increasingly negative direction. The majority of Czech citizens oppose acceptance of any migrants (around 66–67%) and more than 70% oppose allowing refugees to enter the EU and support immediately returning refugees to the country from which they entered the EU. Only 36% would support any permanent solidarity scheme of relocation of refugees within EU states. A policy of border controls for all people, including EU citizens, is supported by 79%, and 50% would agree to help financially and by other means other EU states facing high numbers of refugees (GLOBSEC, 2020).

### Methodology

#### Media content analysis

To answer our research questions, we analysed data from various Czech media outlets across a 4-year period, from 2015 to 2018. The data were downloaded from the Anopress database, which is one of the biggest, most comprehensive and reliable Czech media monitoring services. When it comes to a detailed breakdown of the data, it needs to be underlined that despite the relatively small size of the country, the Czech media market is quite extensive. Consequently, we were forced to narrow the research field. As far as television is concerned, we decided to include the main news programmes of three nationwide broadcasting companies: the public service broadcaster (channels ČT1 and ČT24) and the private firms TV Nova and Prima. For print media, we analysed the following major dailies: MF DNES, Lidové noviny, Hospodářské noviny, Právo (all four are considered quality newspapers and widely read) and Blesk (the most popular tabloid). Finally, for on-line media, we included the following news outlets: idnes.cz, novinky.cz, lidovky.cz, aktualne.cz, ihned.cz, and blesk.cz. It is worth noting that, with the exception of aktualne.cz, all are linked to the print dailies selected for analysis as they share the same owner, with some of the content being web/print exclusive, and some shared. We included in the sample only articles published in the news and commentary sections of the respective servers (we did not analyse blogs or other content which can be found online). Furthermore, while today there are more influential news servers on the Czech market (e.g., Seznam zprávy, Deník N, and Neovlivní, among others), these were established after 2015. Thus, they were deemed irrelevant for the purposes of our study as comparable data for analysis would not be available.

Quantitative, computer-assisted content analysis, which enabled identifications of patterns in the data (Høyland, 2018) followed. In order to proceed with the analysis, relevant articles were identified by a set of primary keywords (and their grammatical variations, since declinations are inherent in the Czech language). This set consisted of the word “migrant” and its synonyms and expressions with similar meanings. After identifying the relevant corpus, we had to identify all relevant frames used in the context of migrants and migration. If frames are understood as individual means of processing and structuring incoming information (Fischer and Johnson, 1986), then it is possible to link mass media coverage and the frameworks individuals employ to interpret events. Media frames have been defined as “a central organizing idea or story line that provides meaning to an unfolding strip of events.” Accordingly, “the frame suggests what the controversy is about, the essence of the issue” (Gamson and Modigliani, 1987, p. 143), or, as Entman (1993, p. 52) put it, “To frame is to select some aspects of perceived reality and make them more salient in a communicating text, in such a way as to promote a particular problem definition, causal interpretation, moral evaluation and/or treatment recommendation for the item described.”

To do so, we decided to take a combined deductive and inductive approach in a back-and-forth, abductive research strategy (Tavory and Timmermans, 2014; Beach and Kaas, 2020). First, we identified the two most-common frames that offered pre-interpretation to the audience by linking the phenomenon of migration with concrete social concepts (Moscovici, 1984). These were the humanitarian frame with its focus on individuals and their needs, often formulated in an emotional or moral way, and the securitization frame driven by the security logic of threats, costs and benefits (Gábor and Messing, 2016). Then, we carried out a pilot study of 300 hundred randomly selected articles from our dataset and identified three more relevant frames for our dataset: EU policies, the migration process, and integration of migrants. The first frame refers to the discussion on relocating migrants and all possible *political* means of migration in the context of the EU. The second frame focuses on describing various aspects of migration as a journey (and includes personal stories about migrating individuals). The last frame refers to the need for integration of migrants into the host societies, and the degree to which they should be integrated.

Thus, we applied a set of secondary and auxiliary keywords to help us understand the frames used in each article and to filter non-relevant texts—e.g., articles on migration of animals, etc.—from the data set. Secondary and auxiliary words were identified based on the pilot study (for further details on concordance analysis, see Kwiecien et al., 2011) of 300 randomly selected articles from our dataset and supplemented with some others that we considered relevant to a particular frame (mostly synonyms and semantically related expressions). In other words, we were looking for expressions that surrounded the main keywords to contextualize meaning and identify the frames used. The full list of keywords can be found in Appendix 1.

This allowed us to compute a score, with each article being a unit of analysis. At this stage, if the selected keywords were found in an article, they were counted. However, to control the variable accounting for the length of the article, each keyword that was present was computed only once. The analysis was broken down into one-year periods to enable an in-depth study of the frames and then integrated to allow for a wider vista. At the end of this part of our research, we were able to identify not only the urgency of the issue in the media, but also the context in which it was presented. Moreover, we were able to observe the changes that occurred over time.

#### Focus groups

In total, we organized four focus groups^1^ with semi-heterogeneous respondents. Each focus group consisted of six to eight respondents and was led by a professional moderator who followed a script covering various aspects of perceptions of migration and related issues: the general image of a migrant in various contexts (moral, security, economic, cultural), personal experiences with migrants, and last but not least perceptions of media coverage of migration and political responses to the challenges posed by migration. The duration of each focus group meeting was roughly 90 min, and the discussions were recorded for subsequent analysis. We believe that the way we constructed our focus groups brought us solid results. Also, the number of sessions was more than sufficient. Patterns in the respondents’ thinking emerged almost immediately, and the level of information saturation after the second session was quite high. We gained no significant new information after this session.

After the data collection, we carried out a detailed analysis. In the first stage, we screened the recordings and identified a set of categories that emerged during the research sessions. This step involved creating a set of mind maps (one for each focus group), which helped us to organize and systematize our further work. These maps allowed us to see the plasticity of the phenomenon and the relationships between various viewpoints and interpretations. After this step was complete, we started looking for direct quotes that would illustrate how our respondents were thinking about the issues. Here, it should be emphasized that due to the qualitative nature of the research, the findings in this section are not representative, and the conclusions cannot be reasonably extended to the entire population of the Czech Republic. Qualifiers used in the analysis, such as “the majority of respondents” or “a small proportion of respondents,” are only descriptive and indicative.

In this paper, we cite the translated statements of the respondents; these are in quotation marks and marked with the number of the interview (B1–B4), whereby B1 corresponds to young responders (<25 years old) living outside Brno; B2 corresponds to young responders (<25 years old) living in Brno; B3 denotes older responders (26+ years old) living outside Brno; and B4 indicates older responders (26+ years old) living in Brno.

### The findings

**Fig. 1:**
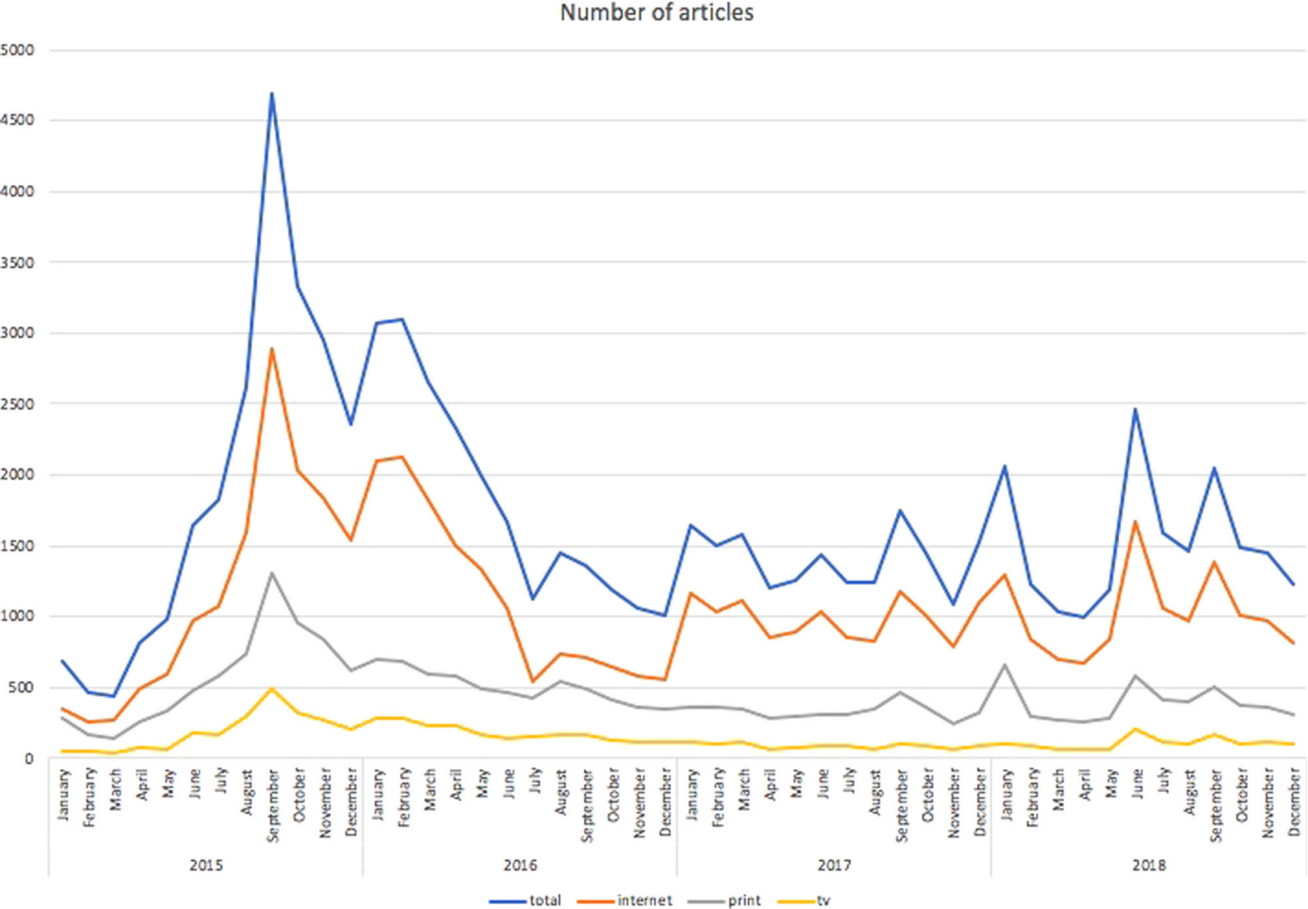
Total number of articles. The total number of articles on migration and migrants in the Czech media 2015–2018.

The quantitative analysis of our data yielded the following results: When it comes to the intensity of the media coverage during the crisis, as shown in Fig. 1 the trend clearly peaked in September 2015 with 4684 appearances in all the media types, including 2894 online articles, 1301 in print and 489 mentions in TV reporting. As we can see from Fig. 1, the coverage did not differ substantially among media types. We can clearly see similar patterns in reporting regardless of media type. Of course, the gross number of published pieces on migration differs, but this is due to the nature of the media. In this context, it is not surprising that the fewest number of pieces were on TV and the most articles were published online.

**Fig. 2:**
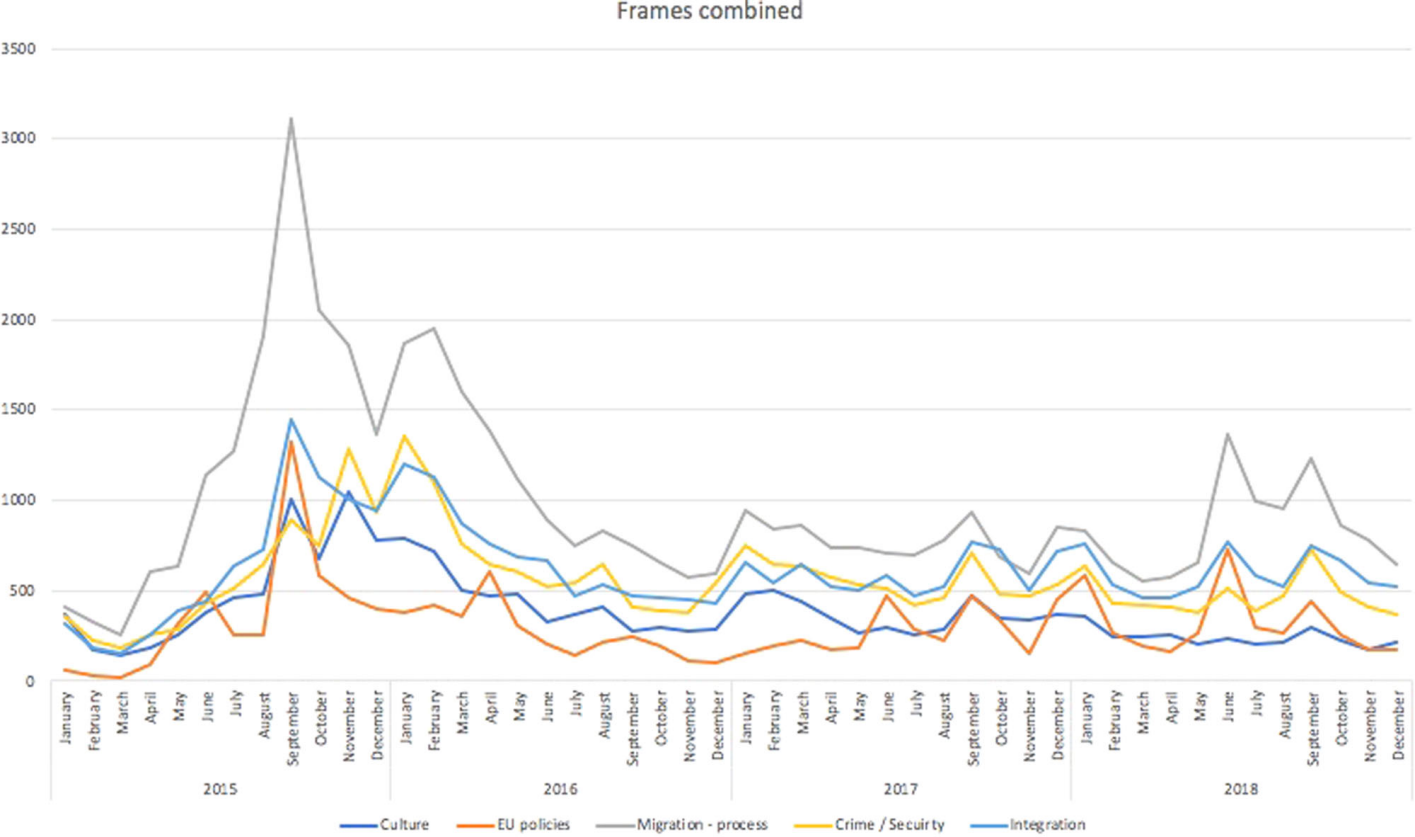
Frames combined. The combined semantic frames used in the Czech media discourse 2015–2018.

To gain a more nuanced understanding of the tone of the coverage, we divided the data according to the leading frame. As we can see in Fig. 2 the intensity of coverage and use of different frames varied over time and it becomes apparent that while coverage of the individual themes was in general synchronized in terms of intensity, the migration process took undisputed precedence and was closely followed by integration-related issues. The trend thus de facto responded to contemporary events in European countries—there was discussion about the need to redistribute refugees among EU countries, and there was a fairly intense debate about cultural differences, integration and security throughout the time period. At the end of the period under review, the debate on cultural differences and EU policies ceased to be significant—the debate at this level ended and quotas ceased to be a topical political issue.

**Fig. 3:**
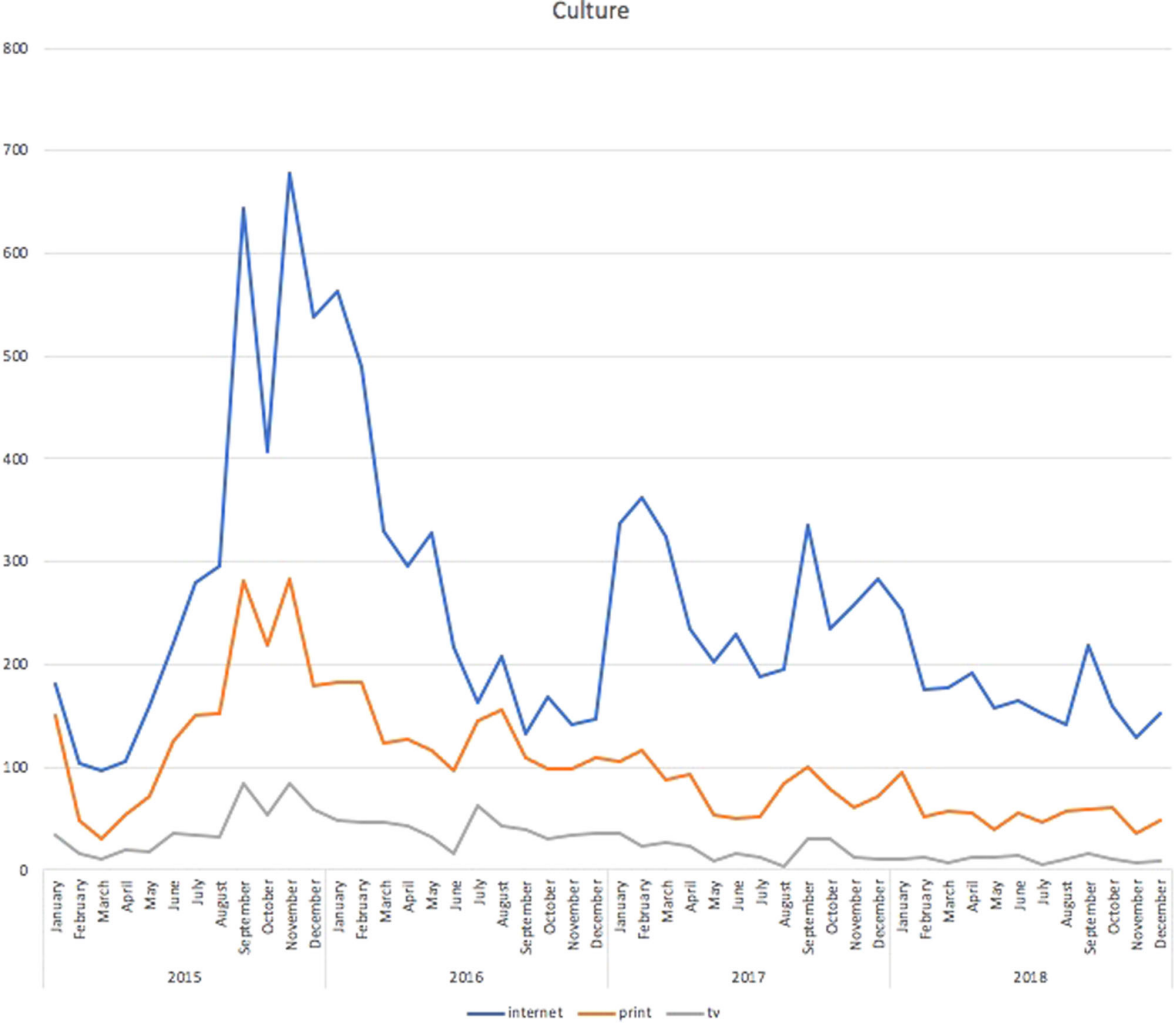
Articles mentioning cultural issues (mostly religion). Cultural frame: Czech media articles mentioning cultural issues (mostly religion) during the period under study.

**Fig. 4:**
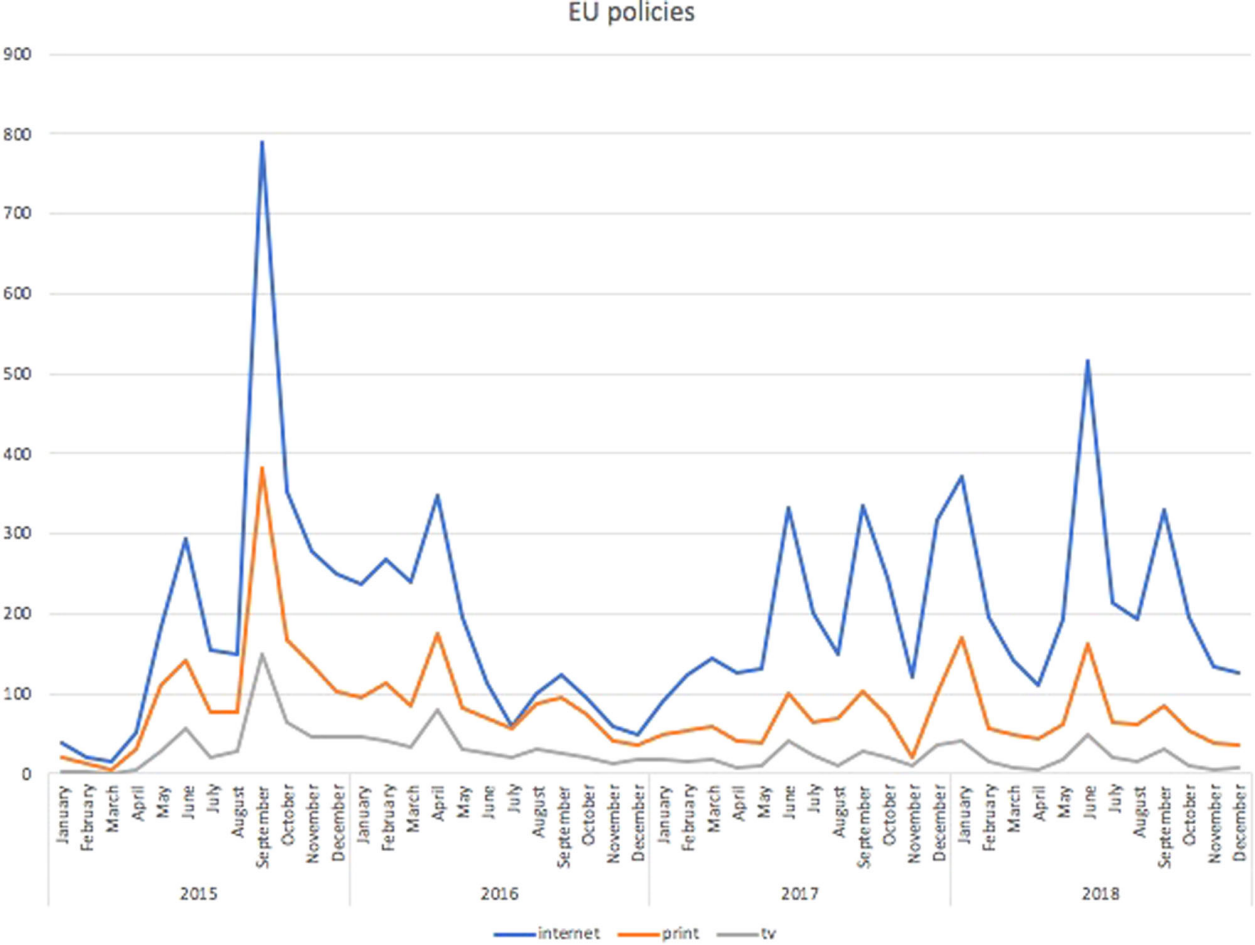
Articles concerning EU policies. Political frame: Czech media articles concerning EU policies during the period under study.

As shown in Fig. 3, the issue of culture was linked to the societal security concept that arose from dealing with a vast influx of immigrants in a relatively short time span, approaching it as a threat to society’s identity. This peaked in sync with the overall intensity of the discourse in September 2015 and then started to decrease. But instead of a systematic decline, Fig. 4 shows that this political frame had regular resurgences.

**Fig. 5:**
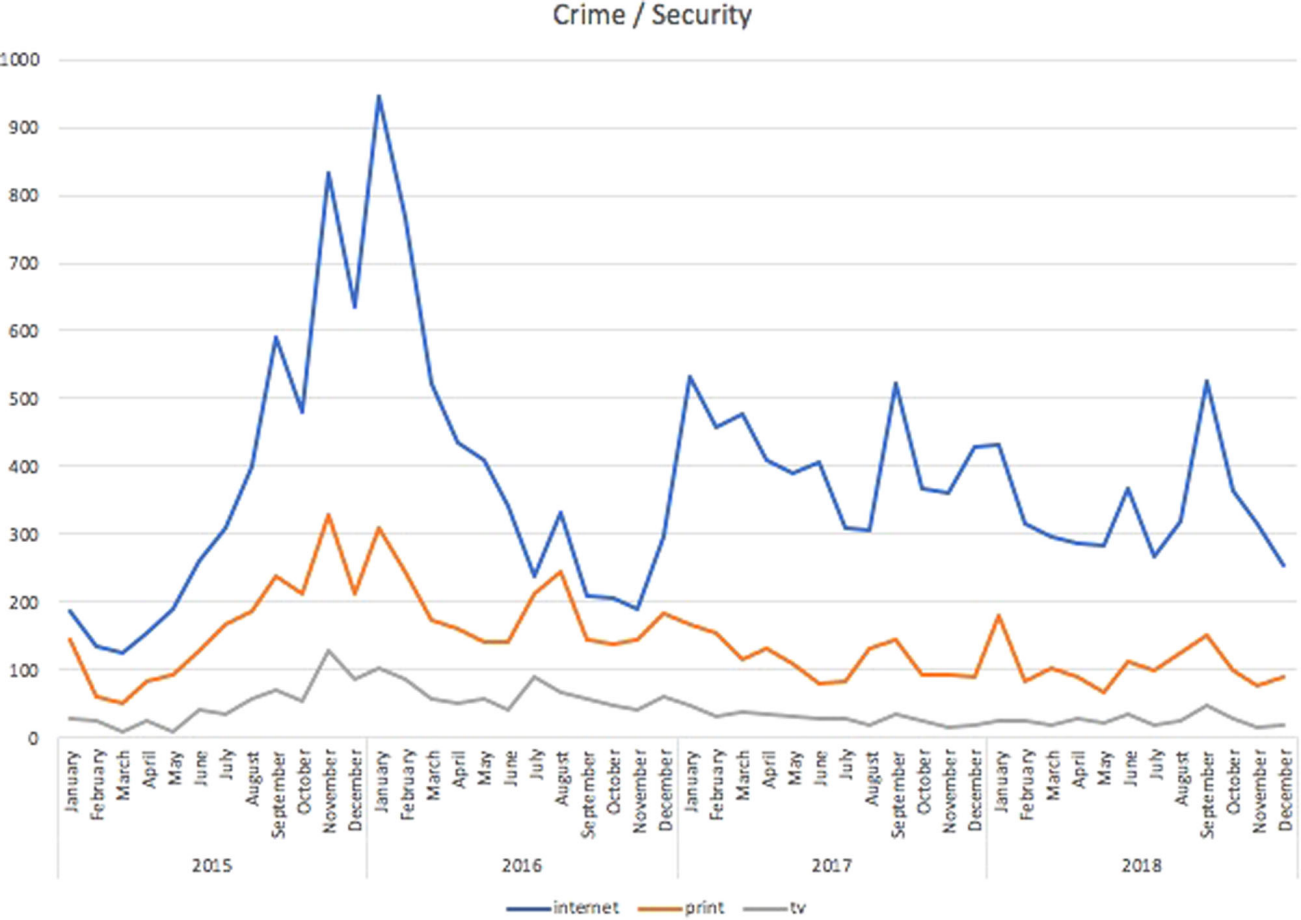
Articles mentioning crime, terrorism and other security issues. Security frame: Czech media articles focused on crime, terrorism and other security issues during the period under study.

There has been a fundamental relationship between the perception of migration processes and media reports of terrorist attacks (Łaciak, Segeš Frelak 2018) (Fig. 5). Unsurprisingly, the media discourse was consistently very negative and selectively focused on terrorism, crime and failed integration. Interestingly, Fig. 5 shows that this frame reached its peak later than the other frames.

**Fig. 6:**
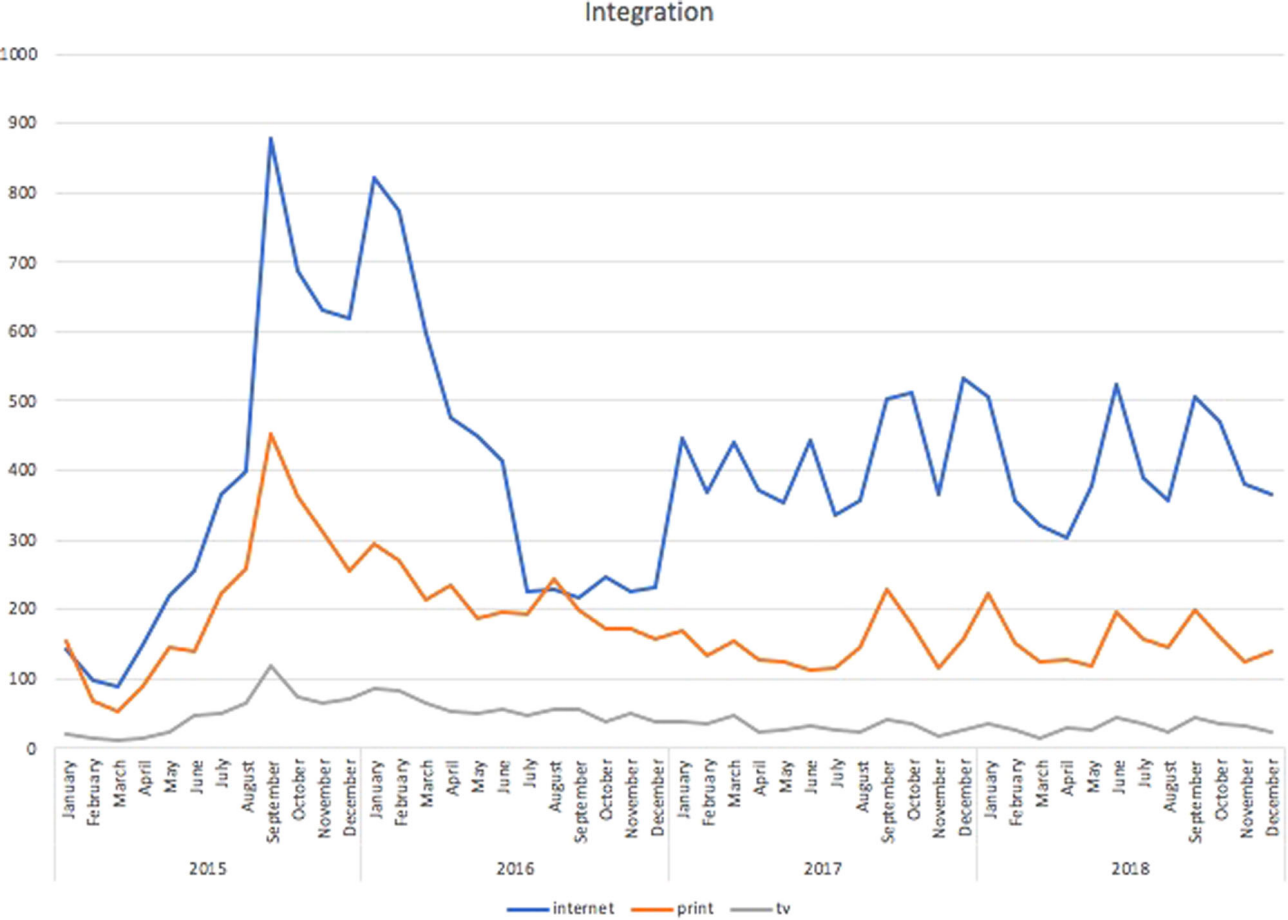
Articles mentioning integration-related issues. Integration frame: Czech media articles mentioning integration-related issues during the period under study.

Given the number of obstacles to integration for non-European immigrants, which greatly hinders their adaptation to the host societies, it is not a surprise that integration was another salient frame in the media discourse. This dimension was also visible in the potency of the cultural motif in the coverage. Unsurprisingly, the two peaks notwithstanding (in September 2015 and February 2016), one can argue that since 2017, with at least nine minor lows (in January, March, July, September, October and December 2017, and January, July, September, and October 2018), this theme was near-constant, as shown in Fig. 6.

**Fig. 7:**
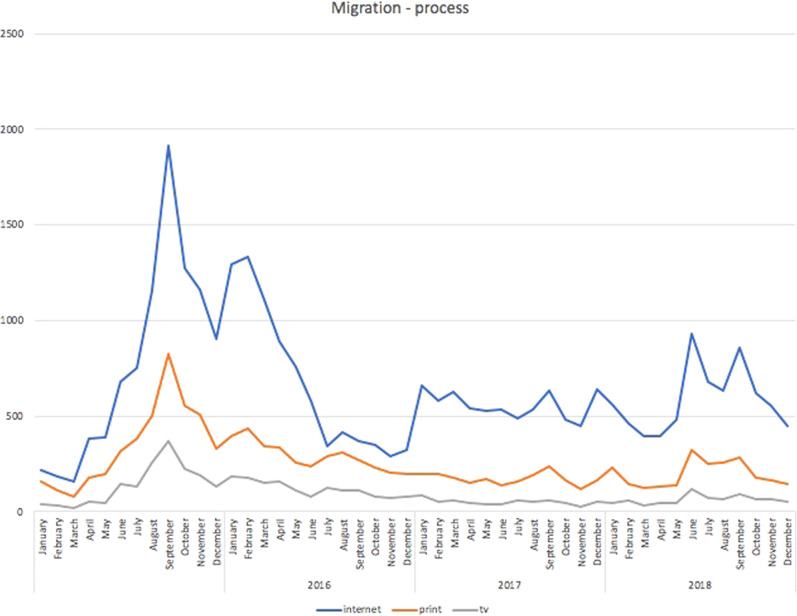
Articles mentioning the migration process. Migration frame: Czech media articles mentioning the migration process during the period under study.

Simultaneously, it is worth noting that, in quantitative terms, the two peaks concerning the integration-related frame would merely constitute a deeper low if we consider the number of articles concentrated on the migration process as a whole. As the most popular frame by far, the migration process received a great deal of attention, with 1916 online articles, 823 in print, and 368 on TV in September 2015, followed by reduced yet sustained interest, above the averages for the other frames as shown in Fig. 7. This is unsurprising as this is a cross-cutting theme that touches upon all three dimensions of the migration issue: the cultural, the political and the economic.

Thus, the issue of migration was covered quite intensely by Czech media outlets, and for a long time it was one of the most salient issues covered by the media. The media reported quite intensely on the process of migration, with journalists focusing on how migrants crossed borders, where they came from, and whether they had to deal with Frontex or national security forces. After the migration process, the media were concerned with the issue of integration of migrants. Very often the media stressed the different cultural or religious backgrounds of migrants. At the same time, the media covered crimes allegedly committed by migrants quite heavily. In other words, the media used the frame of securitization very often. In this respect, they mixed various frames together. The resulting image of migrants was not too positive but matched the tone of the public debate. The next section reveals whether or not Czech citizens shared such rather unflattering attitudes towards migrants.

### The Focus Group interviews

It is one thing to calculate the intensity of the narratives presented in media coverage; it is, however, an altogether different thing to understand the connection between the media messages and the perceptions of migrants and migration among Czech citizens. Our FGIs were meant to bridge the gap between the two. While we are aware that there is a time gap between these two elements of the study, we believe this is not a problem in the context of the interpretative character of the research (cf. Anastasio et al., 1999; Druckman et al., 2012; McCombs et al., 2011). Indeed, the time gap between the media content and its reception allows us to examine the residual effects of framing.

During the four sessions, we first asked participants how they consume media and what they think about it. Then, we discussed migration and their perceptions of migrants. The logic of the script corresponds to the following section.

### The media diet

When it comes to media diet, the FGIs confirmed that most of the respondents preferred online media and the majority of them did not read traditional, printed media. However, they did watch TV and listen to the radio (at least occasionally) and some of them (especially the younger ones) read high quality magazines (e.g., *Respekt*). The reason for choosing on-line media is obvious—according to our respondents, speed, cost and content (online sources have the same or comparable content and quality as other media types) matter most. This was consistent across all age groups, as our respondents offered similar statements: “For younger [people] it is more comfortable. You open your phone and… (B2).” “I got it [news] in my pocket (B4).” “We have everything on our phones (B4).” The convenience of online media was the most visible advantage over other media types. “I wake up and don’t have to go anywhere to buy newspapers (B4).” “I don’t have the feeling of missing something when I’m not buying [printed newspapers] (B2).” “I don’t have time [for reading newspapers]. I go to seznam or novinky, look at the headlines and I’m in the picture (B3).”

Occasionally, some preferred traditional media: “I don’t know why but I trust prints more. The speed is not the most important feature (B1).” Interestingly, for older respondents, rapid proliferation of news was perceived as a weakness of online media. “There is too much information there. News are repeated on various servers (B3).” Nonetheless, the overwhelming consensus was in favour of the Internet. “Prints feel more comfortable. However, I don’t have time to go for them and buy them (B4).” “To read the news on the phone on a tram is much more comfortable [than reading it in printed format] (B4).”

Interestingly, the respondents were aware of the selective exposure and confirmation bias typical for media bubbles: *“*Well, people usually don’t turn TV on and wait until they see what they are looking for (B1).” And thus, it becomes obvious that in general the respondents appreciated the more detailed reporting typical of print media, although this did not outweigh the benefits of online media. On the other hand, social media were not indicated as information sources, or at least were not perceived as such. The traditional media had a good reputation among the audience; they also served as a reference point for the salience and relevance of the news. They were sometimes judged to be more serious. “However, there is too much information/news on the internet. A man must filter to find relevant sources/information. This is a clear disadvantage. I think that in print you can find more salient issues… (B1).” Furthermore, younger respondents pointed to various cognitive biases, especially in social media. “It is important that you can choose what you want. … They hear there their truth; they look there only for what they like (B1).”

In general, we did not detect any significant difference between the respondents’ credibility assessment of online and other media types. However, as shown above, the “traditional” media scored slightly better, even if some of our respondents saw the decline in the quality of traditional media, and vice versa. This sentiment was unanimous across all the interviewed groups. “When the source is credible [e.g., the online version of a print newspaper, etc.], there is no need for fact-checking (B1).” “*T*here are no differences—news are pretty much the same (they tell what happened) (B4).” “It’s the same… The quality of press went down and internet media learned that they have to fact-check (B3).” “A lot of things online is fake news, pointless or useless. In prints everything has been fact-checked (B2).”

Simultaneously, younger respondents distinguished between “alternative” and Institutionalized news outlets. The brand of the publisher was frequently regarded as an indicator of credibility; in this sense the established Czech outlets like *idnes* and *novinky* were juxtaposed with untrustworthy alternative media in general. By the same token, public TV scored better than commercial channels.

Many of the respondents took the news with a grain of salt—they were fully aware of the fact that the published content had an implicit political and economic goal: to attract more attention and give readers what they expect to read. “Journalism today… they don’t lie but they don’t tell the whole truth either. They write only about small pieces, frame it somehow and it is necessary to find the rest (B3).” “The media react to what readers want to hear (B3).” “I have an inner filter activated. I either trust the news (I judge them by my common sense) or not. I believe the media up to 80% (B3).” Thus, media were perceived as very often exaggerating and as favouring negative stories: “News have to be catastrophic. Negativity attracts attention (B4).”

Furthermore, at least the younger respondents understood how the media work, and specifically what agenda setting means. The FGIs demonstrated that this group distinguished between the media’s agenda, where migration was not present, and the political agenda, where migration was used to gain attention or electoral support. This audience was sufficiently media literate to understand the political need for a cover story to distract the public. Their ability to look beyond the smoke screen of a surface narrative was evidenced in how they observed the strength of some political parties (namely the Freedom and Direct Democracy party of Tomio Okamura) that grew precisely by feeding off migration.

In this context, many respondents claimed they did at least some fact-checking of their own, sometimes even verifying the validity of what was reported in foreign media. This happened especially when they found the reports interesting, or they were unsure if the message was plausible. “The media misrepresent reality. If something catches my attention, I look for further information (B4).” “On Instagram, there is only one point of view presented; in the case of TV this is not an issue (B1).”

### Reception of migrants and migration

It is hard to unequivocally conclude whether the respondents believed the migration crisis coverage to be a passing phenomenon or whether they perceived the contemporary challenges concerning migration as a more permanent feature of the news landscape. Migration as such was mentioned by all groups when we asked about current media coverage. However, it was not perceived as one of the most salient topics at that moment. The current political situation in the Czech Republic and global warming/climate change were perceived as much more urgent. It should be noted here that this part of the research took place in early 2020 and the media was slowly starting to focus on Covid-19. The topic of migration was still perceived by the respondents, but in a long-term perspective and it was no longer a priority for them. Therefore, it is difficult to estimate the perceived salience of migration with great certainty. However, the respondents were able to reflect on the process of “fading”: “It depends how it [the issue] unfolds. When there is no development, it [the media] goes silent (B4).” “If it will last for too long (…), no one will care anymore (B4).”

They also perceived a shift in emphasis in the news—they no longer talked about migration as a process, but about migrants and their stories, about how they live in Europe. The media was judged to focus on excesses and sensationalism: “They don’t talk about those who work and pay taxes, that’s a fact. Sex, drugs and scandals sell… (B3)” “When something happens and it gets into the media, it’s okay, it’s objective. But when it’s not said to be one case in a thousand, it’s sensationalism. (B2)”

This lack of priority was further evidenced by the fact that not even one participant in the FGIs said s/he was searching for information on migration on his/her own initiative at that time. The issue itself was perceived (especially by the younger respondents) as salient and unresolved, yet it was not salient enough to push the respondents to look for regular or occasional updates on the subject.

On the other hand, they perceived the topic of migration as important in the political sphere. Indeed, some politicians have regularly used the topic as a tool to activate their supporters and in more than one case the topic of migration has become an election campaign issue (specifically in the 2017 parliamentary elections, and in the 2018 presidential elections). Respondents were aware of this fact and mentioned the topic as a device to easily gain political points and attention: *“*This is an important political issue…. Occasionally something comes up when the government has to deal with something, so they have to define themselves sharply in order to score political points (B2)*”.*

### The mental representation of a migrant

In this section, we will focus on the perception of migrants by Czech citizens. As mentioned above, Czech society is (culturally and ethnically) quite homogeneous. Historically, migrants settling in the Czech Republic integrated almost seamlessly into Czech society and adapted to most of the cultural norms. We need to point out that most immigrants of European origin did not differ (visually) from the host society. This is especially true for migrants from the former Yugoslavian countries and Ukraine. The sizable Vietnamese diaspora community, which used to live somewhat separated from the majority, was not perceived by our respondents in any negative light, as many people from the majority benefit from Vietnamese businesses. The reason for the smooth acceptance of all these groups (despite numerous Balkan and Vietnamese criminal groups operating in the Czech Republic) is their alleged cultural compatibility with Czech society, their hard work, and their willingness to fit in and adapt to local communities and their norms. The latter constitutes a crucial point in the current discussion:

*“*[I know] people from the former Yugoslavia who fled the war. They came and immediately started looking for work and learning Czech. (B3)” In other words, these people have a certain (economical) value for the Czech Republic, they are perceived as migrant second and an economic asset first, and hence do not represent any threat to the host society. This perception goes hand in hand with the idea of an ideal, i.e., controlled migration by the Czech state. Thus, it seems that for most of our respondents, a sense of control over who comes, what they do when admitted, and how they live was important: “The state must be in control. No one can dictate who we accept. (B4)” If respondents did not perceive the economic value or other benefits of migrants, their attitudes were generally negative (although not all of them succumbed to the common media shortcuts and stereotypes): “There is a difference if he is educated and wants to get a job, or if he is an economic migrant and goes purely for benefits with the intention of doing well without a job. (B4)”

However, if asked about the image of migrants on a more abstract level, most of our respondents mentioned spontaneously people from the Middle East or North Africa. In contrast, only a minority of respondents realized that people from EU countries are migrants as well. The reason for this was explained above—people from other regions (from within the EU, the West in general, the Balkans, or the former Soviet republics) are not perceived as significantly different or threatening; on the contrary. Thus, when asked about migrants in 2020, most respondents envisaged an individual (or even a family in some cases) from the Middle East trying to escape war, or (mainly) young men from Africa migrating for economic reasons. Both groups, according to our respondents, flee to Europe for a better life and represent a more or less serious threat to Czech (or even European) society in three linked dimensions: security, culture and economy. The most common argument was that most migrants do not come with good intentions. Within the economic dimension of the issue, the most prevalent attitude was that migrants are attracted to social subsidies. In other words, they have no intention to seek work, and they will rely upon and be dependent on “our” social system (their goal is to “parasite” on the Czech social system): “If only educated and working people will migrate, why not help them? If it’s only those who come for the social benefits and don’t make an effort to integrate, I wouldn’t agree with that. (B4)”

In this context, some of the respondents claimed that the Czech Republic is—*fortunately*—not perceived as a target destination because the Czech state is not so wealthy as Nordic countries or Germany. But at the same time, they feel the need to address the problem in a timely manner: “When it becomes a big issue here, it will be too late. (B4)”

The other two dimensions—culture and security—are very tightly linked. For most of our respondents, migrants, especially those from Islamic countries, represent a serious threat. They come from entirely different cultural and religious backgrounds and do not intend to give up their way of life, their traditions or values. Moreover, they seek a new life within more or less closed communities; new ghettos and socially excluded localities may arise (for a detailed overview of the future-oriented threat dynamic please see Bartoszewicz et al., 2022). With the voluntary isolation of the newcomers come the threats of radicalization, crime, violence, and terror (as seen in some Western countries): “Integration is only possible to a certain extent (depending on their numbers) (B1)” “if there are no clear rules (for integration), there may be some change (which we don’t want) (B4).”

Although the general image of a migrant is largely negative, a minority of our respondents expressed understanding and even compassion for migrants as people in need who are made vulnerable by the geopolitical situation, climate change, or war. For these respondents, migrants were perceived not in terms of their alleged intentions but their personal problems—they were seen as abandoned, poor and hungry mothers (parents) with small kids. Some of the migrants were also perceived as victims of human traffickers, and were moved by stories about their life in Europe. However, even realizing this dimension of the issue did not lead to an accepting attitude toward these people: for these respondents, asylum in the Czech Republic, if it is granted at all, should be only temporary. Once the cause of the displacement (e.g., war or conflict) is resolved, the migrants should return to their home countries. Thus, in most cases, migrants—regardless of their reasons for entering Europe—still represented a more or less serious threat to Czech society.

**Fig. 8:**
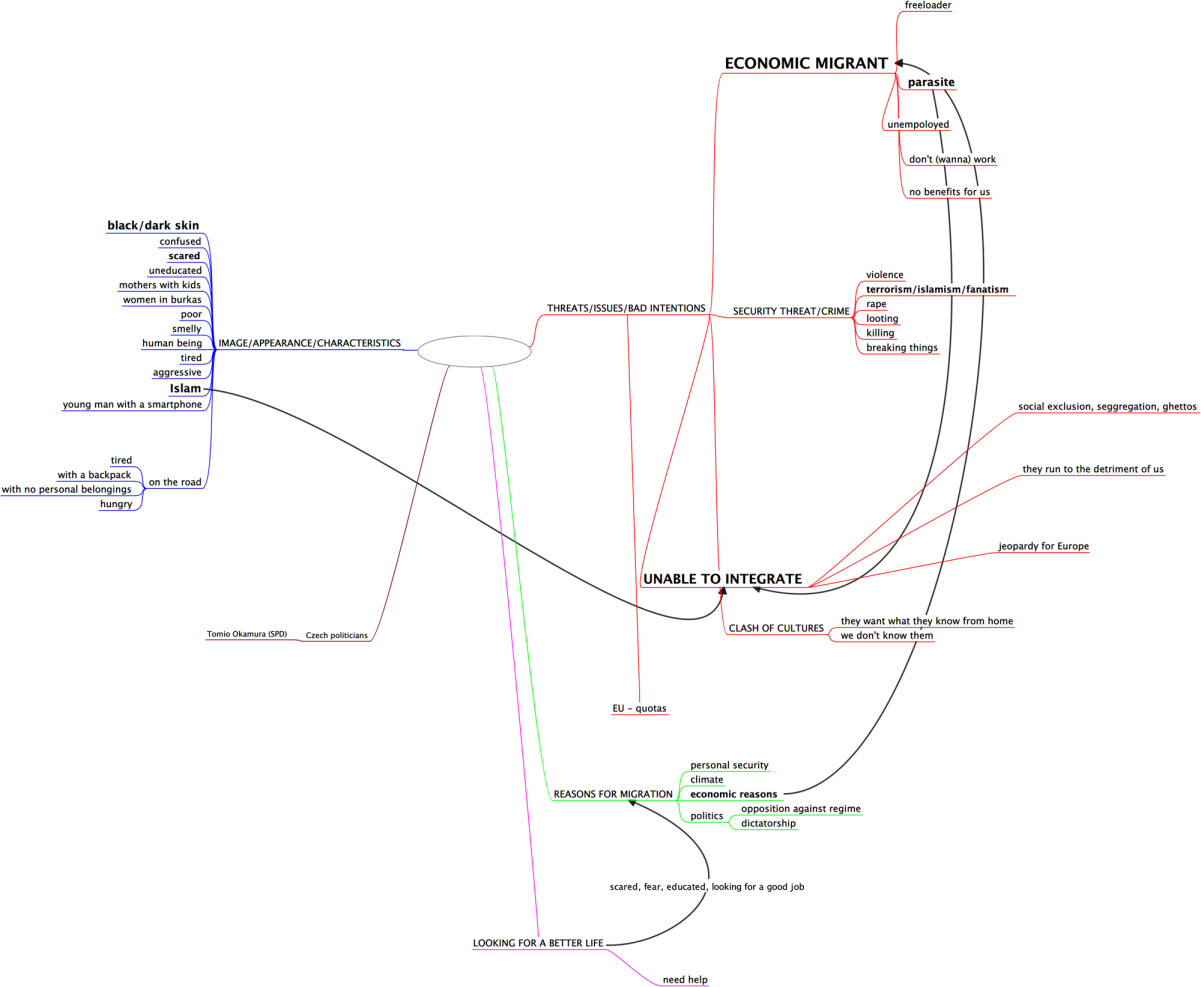
Mind-map representing the mental image of the migrant. Visualisation of the mental mindmap of a migrant developed by O. Eibl on the basis of focus group interviews carried out during the study.

To conclude, the overall image of the migrant can be summarized by the following mind-map (Fig. 8), which on the one hand contains most of the media frames, but on the other, places the accents differently, emphasizing the economic dimension and the inability to integrate as the two most significant tropes.

## Discussion

The existing studies suggest that media discourse is a crucial factor for the societal reception of migration, although the exact findings of various studies differ, depending on their research design and scope (Iyengar, 1991). Héricourt and Spielvogel (2013) find media exposure to be a key determinant of popular beliefs about the influence of immigration on the economy. A study of the effects of media representation of immigration on the perceptions and emotions of citizens in Italy and Spain shows that news frames indirectly affect support for anti-immigration policies (López-Rodríguez et al., 2020). Similarly, van Klingeren et al. (2015), on the basis of their study on the Netherlands and Denmark, cautiously conclude that the longevity of the issue’s salience has a moderating effect on the media impact, insisting that a positive tone in coverage reduces popular negativity towards immigration, while a negative tone does not increase popular negativity. In general, the majority of studies show that exposure to negative or positive media messages about immigrants leads to respective negative or positive attitudes toward migration and mobility among media consumers (Meltzer et al., 2017). A comparative content analysis of migration coverage in 22 opinion-leading newspapers in six European and five sub-Saharan African countries found that European coverage of the 2015 crisis was dominated by domestic issues (Fengler et al., 2020). Specifically in the Czech context, Urbanikova and Tkaczyk (2020) found that the media framed the refugees and migrants mainly as a burden on the host society, a finding that has been corroborated in our FGIs.

The pragmatic, order-inclined nature of the Czechs, consistent with their heterostereotype articulated by other national groups (Hřebíčková and Graf, 2014; Hřebíčková et al., 2017), is evident in how the practicalities of the migration process and the integration-centred frames dominated the Czech media discourse. The Czech media focused on how migrants come, which was later reiterated during the FGIs with statements referring to state control. Furthermore, the forward thinking of “they come and what then?” is attested by the preoccupation of Czech media with integrating migrants into Czech society. The questions of what migrants do after arrival and how they live suggest the existence of a structural relation between both groups. Linkages between migrant labour and host societies evolved, according to Arango (2000), as a part of an inevitable global capitalist process. The countries of Wallerstein’s (1974) peripheries have at their disposal economically uprooted men and women, ready to migrate to take advantage of opportunities in the wealthy urban cores of the world (Massey, 1999, p. 42), where the need for immigrant workers is apparent.

This brings us to the most interesting insight gleaned from our research, namely the markers of what makes one a migrant, or the answer to the question, “why are only some people who are mobile across space called ‘migrants’ while others are not?” (Schweppe and Sharma, 2015). To account for the opaqueness of the concept, Charsley and Wray (2015) go so far as to claim migrant invisibility in certain circumstances. The research devoted to the image of the migrant, that is to say, on either the visual representation or social imagery of the migrant figure, focuses mostly on cultural representations (Lutz, 1991; Burçoglu, 1999; Demos, 2013; de Oliveira, 2014; Nail, 2015) as these are normally put forward as primary differentiators. However, everything that concerns aesthetics and politics (Rancière, 2004) renders the “the migrant” a generic figure (Fortier and Lewis, 2006) that must be broken down to an array of subtypes. These are created through adjectives, and thus we have forced, labour, temporary or illegal migrant. The migrant as a labourer is a fairly common picture (Stahl, 1995; Silvey and Lawson, 1999), although the attitudes to the so-called economic migrants pave way to the most negative image, especially on social media (Komarova, 2020).

Against prevalent images of labour migration and the associated meanings attributed to it, Nail (2015) in particular re-theorizes the migrant from the perspective of motion, rejecting the place-bound membership category. To put it simply, just going somewhere does not render one a migrant. In the construction of the social category of a migrant, law is often the guide to discern between those varying incarnations (De Haas, 2010). The emphasis in definitions of foreign migrant workers from the International Labour Organization, the International Organization for Migration, and the United Nations, as persons whose citizenship is different to the country of current residence, is an important element in further comprehension of who is a migrant.

Indeed, definitions of “migrant” vary from someone foreign-born to someone who has temporarily moved to another country. In the public debate, the term is loose and very often conflates different societal, economic and political issues, which is challenging for policy-making since not every migrant is subject to state control and legislation (Anderson and Blinder, 2015). In terms of what makes one a migrant—be it nationality, place of birth, reason for coming or duration of stay—all might differ. Interestingly, in the Czech case, the majority of migrants, and specifically those with residence permits for employment, family reunification, study, or business, were not labelled as migrants by our FGIs respondents.

The social production of the migrant in the Czech context has an interesting twist in this regard. Working and contributing to the society removes one from the migrant category, and cultural affinity or the specific legal terms of the stay are of secondary importance here. For Czechs, people who come to settle and work are excluded from the migrant imagery. Anecdotally, one of the researchers conducting the study was a foreign-born national of another country working in the Czech Republic and living there with her family. Yet, she was not considered a migrant.

This obviously influences the public’s understanding of migration policy debates, thus imprinting heterogeneous visual symbols onto the political fabric if we agree that symbolic and discursive practices play significant roles in constituting the material world. Also, a warning comes from group theory, which assumes that groups compete for scarce resources (Bobo and Hutchings, 1996). This dynamic makes immigrants competitors in a static market and makes the goods they receive unavailable to the autochthonous population. Furthermore, at the societal level, immigrants pose a risk to taxpayers and the general social welfare system, as they need access to healthcare, settlement services etc. (Aalberg et al., 2012; Facchini and Mayda, 2008; Funk, 2000; Hainmueller and Hiscox, 2010). Our findings confirm that such negative sentiments are already ripe in the Czech society (see Fig. 8).

## Conclusions

Our study shows that despite the increasing numbers of migrants living in the Czech Republic, the general sentiment towards immigration is rather negative and depends on how “migration” is portrayed in the media, how it is handled by (political) elites and how it is understood by the society at large. The crucial factor in assessing the whole issue is the fact that citizens and political representatives alike lack significant (personal) experience with migration, and while the actual situation is quite calm, the public debate is not. Therefore, one might argue that the Czech case is an acute scenario whereby wild imagery presented by the media shaped the perceptions and the attitudes of the audiences and amplified some pre-existing stereotypes.

The media coverage of migration between 2015 and 2018 was quite intense, regardless of media type, and journalists used various frames and contexts when reporting about migrants and migration in general. Thousands of articles with rather negative frames were published and most Czech citizens were exposed to that content on a daily basis. The issue of migration became a main focus of many political campaigns, was used as a mobilization tool for populist and extremist parties, and influenced significantly the Czech political landscape (even though there were literally no migrants seeking for asylum in the Czech Republic at the time).

Interestingly, our respondents were aware of how exaggerated and inflated the news coverage was. They frequently commented on scandalization in all respects and contexts, and the focus on negativity (crimes, scandals) with almost no positive reporting in the media. In their view, the main goal behind such an angle is purely utilitarian, as the media aim to provoke fear to gain more attention, which brings more readers, and therefore more income.

In this regard, the respondents also noticed that the media discourse was not so much about migration, but rather migrants. In doing so, the media was juxtaposing personal stories with a big threat. Regardless of framing, the motive of fear was omnipresent and reinforced and supported by the numbers of migrants—the more migrants, the bigger the fear or doubts. The younger respondents also saw differences in the styles of reporting, as they were able to distinguish between fear mongering and evidence-based reports. Interestingly, although the older respondents did not possess similar capabilities, they did not trust the media image of migration and felt that there was something hidden from them. It was our conclusion that the older audience more often than the younger respondents relied on their own common sense. Sometimes members of the older age groups expressed frustration that they were overwhelmed with information and did not know what to think, or what was really happening. Since they were, by their own admission, confused, we were not surprised to see that their lack of information resulted in repeating phrases or stereotypes learned from the media.

## Supplementary information

Appendix 1

## Data Availability

The datasets used and analysed during the current study are available from the corresponding author on reasonable request.

## Footnotes

1 The research took place on 3 and 14 February 2020. A market research agency helped with recruitment. The recruitment criteria were simple: we decided to control only age (young vs. old respondents), place of residence (big vs. small cities or villages) and level of education (all of our respondents had either secondary or higher education). Thus, we deliberately did not control for gender, income, political preferences, or even their initial attitudes towards migration or their personal experience with migrants or migration. The reason was that we sought to assess the “whole” picture and were not interested in isolated pro- or anti-migrant discussions. Thus, two groups consisted of young and older respondents from a bigger city, and the other groups of those from villages (or smaller cities).
