# Supplementary material for: A rather wild imagination: who is and who is not a migrant in the Czech media and society?
Source: Humanit Soc Sci Commun. 2022 Jul 6;9(1):230. doi: 10.1057/s41599-022-01240-2 (PMC9258767; doi:10.1057/s41599-022-01240-2)
Supplement: Supplementary file 1 — Appendix 1 [file 41599_2022_1240_MOESM1_ESM.docx]

Appendix 1

Keywords selected for content analysis:

**Main keywords**

| migrant |
| --- |
| uprchlík |
| běženec |
| přistěhovalec |
| azylant |
| utečenec |
| migrace |
| celkem skore |
| norm score |
|  |
| Amerika |
| USA |
| Kanada |
| Mexiko |
| Latinská |
| karavana |
| celkem |
| norm score |
|  |
| Evropa |
| EU |
| Afrika |
| Asie |
| Blízký Východ |
| Schengen |
| Visegrád |
| celkem |
| norm score |
|  |
| Británie |
| Francie |
| Německo |
| Belgie |
| Nizozemsko |
| Rakousko |
| Dánsko |
| Norsko |
| Švédsko |
| Finsko |
|  |
| Turecko |
| Řecko |
| Makedonie |
| Bulharsko |
| Rumunsko |
| Srbsko |
| Chorvatsko |
| Bosna |
| Slovinsko |
| Maďarsko |
| Balkán |
| Slovensko |
| Itálie |
| Sicílie |
| Sardínie |
| Korsika |
| Kypr |
| Kréta |
| Španělsko |
| Malta |
| Česká republika |
| Jordánsko |
| Libanon |
|  |
| Ukrajina |
| Sýrie |
| Irák |
| Írán |
| Egypt |
| Sudán |
| Eritrea |
| Etiopie |
| Libye |
| Alžírsko |
| Nigérie |
| Kongo |
| Afghánistán |
| Pákistán |
| Bangladéš |

1. **Culture/Religion**

| muslim |
| --- |
| islám |
| mešita |
| Mohamed |
| Aláh |
| Islámský stát |
| křesťanství |
| Ježíš |
| Žid |
| celkem |
| norm score |

1. **EU policies**

| kvóty |
| --- |
| fond |
| promigrační |
| protimigrační |
| celkem |
| norm score |
|  |
| brexit |
|  |
| Miloš Zeman |
| Andrej Babiš |
| Bohuslav Sobotka |
| Vojtěch Filip |
| Karel Schwarzenberg |
| Tomio Okamura |
| Pavel Bělobrádek |
| Miroslava Němcová |
| Petr Fiala |
| Radim Fiala |
| Marek Černoch |
| Jan Hamáček |
| Lubomír Zaorálek |
| Miroslav Kalousek |
| Jiří Pospíšil |
| Ivan Bartoš |
| Jan Farský |
| Petr Gazdík |
| Dita Charanzová |
| Martina Dlabajová |
| Petr Ježek |
| Jan Keller |
| Jaromír Kohlíček |
| Kateřina Konečná |
| Petr Mach |
| Jiří Maštálka |
| Luděk Niedermayer |
| Jiří Payne |
| Pavel Poc |
| Miroslav Poche |
| Stanislav Polčák |
| Miloslav Ransdorf |
| Olga Sehnalová |
| Pavel Svoboda |
| Michaela Šojdrová |
| Jaromír Štětina |
| Pavel Telička |
| Evžen Tošenovský |
| Tomáš Zdechovský |
| Jan Zahradil |
| Martin Konvička |
| Tomáš Vandas |
| Petr Hampl |
|  |
| Donald Tusk |
| Jean-Claude Juncker |
| Johannes Hahn |
| Federica Mogherini |
| Viktor Orbán |
| Geert Wilders |
| Recep Tayyip Erdoğan |
| Heinz-Ch. Strache |
| Norbert Hofer |
| Marine Le Pen |
| Angela Merkelová |
| Emmanuel Macron |
| François Hollande |
| Alexis Tsipras |
| Giuseppe Conte |
| Paolo Gentiloni |
| Matteo Renzi |
| Mariano Rajoy |
| Pedro Sánchez |

1. **Migration process**

| Frontex |
| --- |
| člun |
| loď |
| moře |
| vlak |
| záchrana |
| uprchlické centrum |
| uprchlický tábor |
| detence |
| detenční centrum |
| převaděč |
| pašerák |
| policie |
| celník |
| pohraničník |
| voják |
| armáda |
| kontrola |
| bariéra |
| zeď |
| plot |
| chodba |
| podzemní |
| celkem |
| norm score |

1. **Crime/security**

| terorismus |
| --- |
| terorista |
| obtěžování |
| kriminalita |
| sex |
| znásilnění |
| krádež |
| vražda |
| zbraň |
| diskriminace |
| útok |
| přepadení |
| napadení |
| celkem |
| norm score |

1. **Integration**

| integrace |
| --- |
| multikulturalismus |
| adaptace |
| přizpůsobení |
| kultura |
| tradice |
| jazyk |
| práce |
| učení |
| celkem |
| norm score |

1. **Type of migration**

| nelegální |
| --- |
| ekonomická |
| environmentální |
| válka |
| válečný |
| humanitární |
| diktátor |
| celkem |
| norm score |
| war |
| warnorm |
